# Migration of Distal End of VP Shunt into the Scrotum: A Management Review

**DOI:** 10.1055/s-0042-1756181

**Published:** 2022-09-02

**Authors:** Mahmoud M. Taha, Hassan A. Almenshawy, Mohammad Ezzat, Mohamed Kh. Elbadawy

**Affiliations:** 1 Department of Neurosurgery, Zagazig University, Zagazig, Egypt; 2 Department of Neurosurgery, Al Mokatam Insurance Hospitals, Cairo, Egypt

**Keywords:** VP shunt, migration, scrotal swelling, hydrocele

## Abstract

Ventriculo-peritoneal (VP) shunt is the typical and most common procedure for the treatment of hydrocephalus. Many complications have been associated with VP shunts, migration of the distal end of the VP tube into the scrotum is a rare one. We report the presentation and management of a case of 3 month age infant who had scrotal swelling primarily diagnosed as hydrocele. Investigations proved the presence of shunt migration. The possibility of shunt migration should be considered. Early diagnosis and management of such complications is easy and can prevent subsequent serious sequelae.

Ventriculo-peritoneal (VP) shunt is the first option procedure for the treatment cases with hydrocephalus. Such a procedure is associated with a wide variety of reported complications related to the entire shunt system. 1 One of these complications is shunt migration, with an uncommon occurrence in one/1,000 cases who undergo a shunt procedure. 2 It is crucial to early diagnose and treat these uncommon problems. 2 Distal shunt migration into the scrotum combined with or as a result of inguinal hernia through patent processes vaginalis can result in more significant consequences, including scrotal edema, acute scrotum, and shunt extrusion. 3 Here we represent a case with scrotal migration of the peritoneal end of the VP shunt system.

## Case

**Fig. 1:**
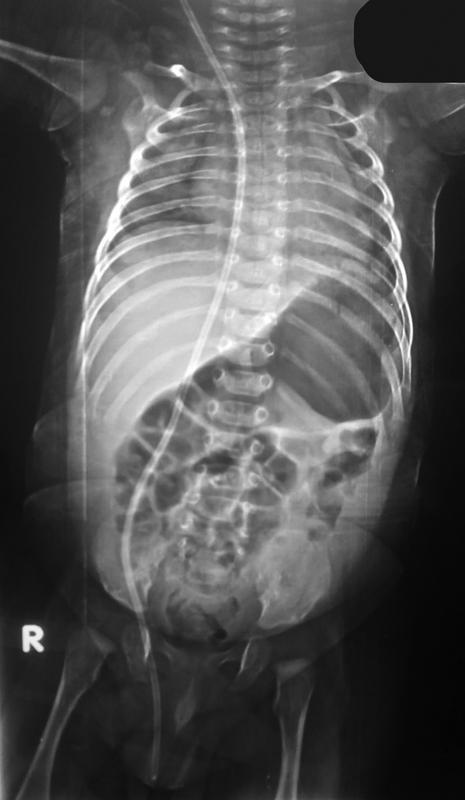
Plain X-ray of abdomen anteroposterior view, showing tip of peritoneal end of the shunt located in the right scrotum.

**Fig. 2:**
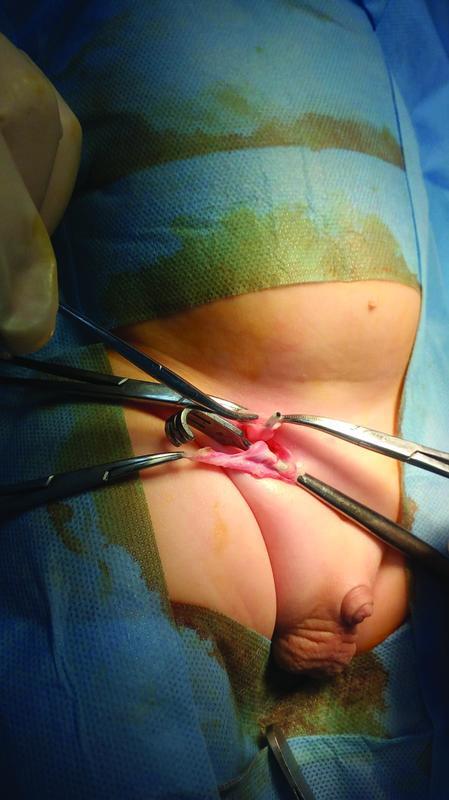
Intraoperative image, showing the incision at inguinal ligament and tip of the shunt before replacement.

A 3-month-old infant was presented by his mother with scrotal swelling for 1 week. The mother gave a history of surgery for repair of myelomeningocele 1 week after birth, and 3 weeks later he developed hydrocephalus and was operated on for insertion of VP shunt. The surgeon used Medtronic VP shunt (burr-hole type, medium pressure). The ventricular end was placed through Frazier burr hole, and peritoneal end through right subcostal incision. By examination, there was a reducible scrotal swelling with a palpable tube inside the scrotum and a positive transillumination test. There were no signs or symptoms of inflammation, and the shunt was functioning. Investigations were done. Plain X-rays of the abdomen and pelvis ( Fig. 1 ) showed the distal end of the tube located in the right scrotum. Pelvic-abdominal ultrasound confirmed the diagnosis of shunt migration into the scrotum, with the absence of abnormal masses. Displacement of the catheter was not possible through manual compression. So patient was prepared for surgical repositioning of the migrating tube. Exploratory laparotomy was performed. Through an incision at the right inguinal area, the inguinal ring was reached. The shunt tube was withdrawn out from the scrotum ( Fig. 2 ) with egression of the collected CSF. CSF was seen clear. Distal end of the shunt was replaced in the peritoneum again under direct vision.

**Fig. 3:**
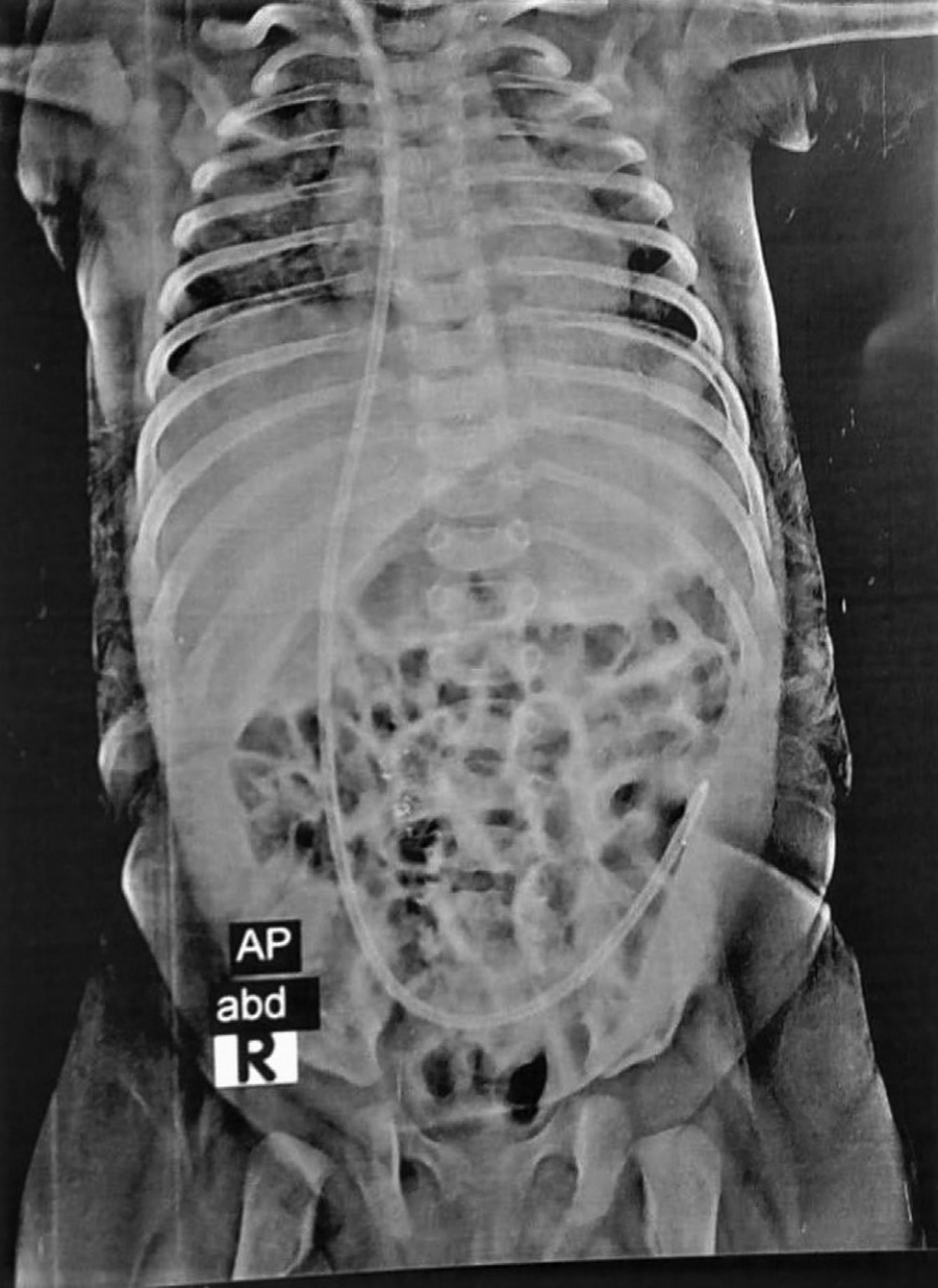
Postoperative plain X-ray of abdomen and pelvis showing the distal tube properly replaced.

The pediatric surgeon performed prophylactic obliteration of processus vaginalis to prevent recurrence. Postoperative plain X-ray of the abdomen ( Fig. 3 ) and pelvic-abdominal ultrasound confirmed the proper position of the distal end. After 6 months of follow-up, the patient was asymptomatic without recurrence or further complications.

## Discussion

Migration of the distal end of the VP shunt into the scrotum through patent processus vaginalis (PPV) is a rare complication. Up to the best of our knowledge, the exact incidence of this complication is not determined in the literature to date. This may be attributed to the rare occurrence of such complication. 1 However, some articles reported the incidence of migration of distal end of VP shunt to be 10%. In a study on 108 pediatric cases with VP shunt, the incidence of scrotal migration was found to be 3.7%. 4

The processus vaginalis is an evagination of the peritoneal cavity through the inguinal canal, it forms during embryologic development in both sexes. In males, the testes migrate from the abdomen to the internal inguinal ligament during the 28 ^th^ week of gestation, and enter the scrotum by the 32 ^nd^ week of gestation. In females, the round ligament of the uterus passes through the inguinal ligament and terminates in labia majora. A PPV persists when it fails to close. 5

PPV is present in 90% of males at birth, 50% at 1 year, 40% in childhood years and 15 to 30% in adulthood. 6 In the presented case, the age at presentation was 3 months.

Scrotal migration of the VP shunt can cause secondary hydrocoele and shunt malfunction leading to worsening of the hydrocephalus. 4 7 In our case the shunt was still functioning without worsening of hydrocephalus, this may be due to early presentation and absence of inflammation.

Although scrotal migration of shunt is not a very threatening complication, but some authors reported serious complications like an acute scrotum, 7 incarcerated hernia (high chances in infants and younger children), 8 which can be confused with a para-testicular tumor, 1 with scrotal perforation. 9 Early diagnosis and management can preclude these complications.

Many theories had been postulated in the literature to explain the mechanism of scrotal migration of VP shunt. The most accepted mechanism is believed to be the increased intra-abdominal pressure leading to increased incidence of inguinal hernias accompanied by the peritoneal end of VP shunt. 10

Many factors are attributed to the occurrence of this complication, the elevated intra-peritoneal pressure caused by continuous CSF drainage from the VP shunt and by the relatively smaller peritoneal cavity in young children, the presence of PPV which is also maintained by the elevated pressure. 1 These factors helped in the formation of clinical hernia and subsequent migration of the distal tube end.

Scrotal migration in cases with multiple shunt tubes had been reported to our knowledge in two cases. 5 11 In one of them the author advised laparoscopic search for PPV or occult hernia in the first VP surgery, and prophylactic repair if present. 5

Other superimposed factors in children with other illnesses, children with meningomyelocele as in our case, include the low absorbing capacity of the peritoneum, weak abdominal muscles, or raised abdominal pressure after the repair of meningomyelocele. 1 8

Many modifiable factors are reported to increase the risk of shunt migration, such as the placement of a long peritoneal tube to avoid future tube change with child growth. Therefore some surgeons advised shortening of the peritoneal end in cases of suspected PPV. 12 Also, the cases where a low-pressure shunt is used lead to excess CSF drainage. 1

The aim of surgery in these cases includes reinsertion of the catheter inside the peritoneal cavity and prevention of recurrence by proper closure of PPV. 4 In most cases, the shunt is functioning well, otherwise, complete shunt revision is required (presence of infection or obstruction). 13

In early infancy, PPV is mainly bilateral (75–80%). 10 Therefore, in the repair of PPV, some surgeons have advised closure of contralateral PPV as a preventive measure for the recurrence. 7

Laparoscopic repair of scrotal migration of the shunt presents many advantages rather than being a minimally invasive procedure, including the replacement of distal end properly inside the peritoneal cavity, especially with the presence of adhesions from previous recurrences, and the ability to explore and close contralateral PPV. 1

In the follow-up, we did frequent checks for any recurrence of palpable hernias or scrotal swelling. This has also been recommended by some authors as a routine follow-up and screening after distal catheter replacement in cases of scrotal migration. 5

## Conclusion

Scrotal migration of shunt is a rare but benign complication after VP shunt surgery in pediatric patients. Pediatric surgeons should be consulted to repair the associated hernias and close PPV to avoid recurrence. We recommend that all infants presented with scrotal swelling with a history of VP shunt should have a neurosurgical examination. Early surgical intervention needs to be taken into consideration.
